# Tracing of Di-Ethylhexyl Phthalate in the Tequila Production Process

**DOI:** 10.3390/foods13020334

**Published:** 2024-01-20

**Authors:** Jose Tomas Ornelas-Salas, Juan Carlos Tapia-Picazo, Antonio De Leon-Rodriguez

**Affiliations:** 1 Tecnológico Nacional de México-Instituto Tecnológico de Aguascalientes, Departamento de Ingeniería Química, Av. Adolfo López Mateos 1801, Ote. Fracc. Bona Gens, Aguascalientes C.P. 20256, Ags., Mexico; tornelas@edu.uag.mx (J.T.O.-S.); juan.tp@aguascalientes.tecnm.mx (J.C.T.-P.); 2 Maestría en Procesos del Tequila, Universidad Autónoma de Guadalajara, Av. Patria 1201, Lomas del Valle 3ª Sección, Zapopan C.P. 45129, Jal., Mexico; 3 División de Biología Molecular, Instituto Potosino de Investigación Científica y Tecnológica, Camino a la Presa San José 2055, San Luis Potosí C.P. 78216, SLP, Mexico

**Keywords:** phthalates, alcoholic beverages, endocrine disruptors

## Abstract

The purpose of this study was to determine the origin, presence, and fate of the endocrine disruptor di-ethylhexil phthalate (DEHP) during tequila production. For this, three tequila factories (small, medium, and large) were monitored. DEHP concentrations in water, agave, additives, lubricating greases, neoprene seals, and materials of each stage process were analyzed using gas chromatography/mass spectrometry. DEHP mass balances were performed to identify the processes with significant changes in the inputs/outputs. DEHP was detected in agave at up to 0.08 ± 0.03 mg kg^−1^, water 0.02 ± 0.01 mg kg^−1^, lubricant greases 131.05 ± 2.80 mg kg^−1^, and neoprene seals 369.11 ± 22.52 mg kg^−1^. Whereas, tequila produced in the large, medium, and small factories contained 0.05 ± 0.01, 0.24 ± 0.04, and 1.43 ± 0.48 mg kg^−1^ DEHP, respectively. Furthermore, in waste materials (vinasses and bagasse) released, 534.26 ± 349.02, 947.18 ± 65.84, and 5222.60 ± 2836.94 mg of DEHP was detected for every 1000 L of tequila produced. The most significant increase in DEHP occurred during the sugar extraction and distillation stages. Results demonstrate that main raw materials, such as agave and water, contain DEHP, but lubricant greases and neoprene seals are the major sources of DEHP contamination. Identification of the contamination sources can help the tequila industry to take actions to reduce it, protect consumer health and the environment, and prevent circular contamination.

## 1. Introduction

Tequila is one of the most consumed spirit beverages in the world. It is the product of the distillation of fermented must from the hydrolysate polysaccharides of *Agave tequilana* Weber var. *azul*. The beverage is produced in factories located exclusively in specific regions with origin denomination, protected by the Official Mexican Standard [1,2]. Tequila factories are classified according to the liters of tequila produced per year as micro (less than 0.3 million), small (from 0.3 to 1 million), medium (from 1 to 3 million), and large (more than 3 million). Each factory has particular operational conditions, but stages of tequila production can be summarized as hydrolysis of inulin, extraction or milling, fermentation, distilling, and filtration.

The main problems in the alcoholic and non-alcoholic beverage industry are chemical, microbiological, and physical hazards. Since food safety is crucial, if good manufacturing practices are not properly followed, the processing of raw materials can represent a major source of contamination [3]. Possible chemical contamination in distilled alcoholic beverages includes metals like copper, and organic substances such as carbamates, microplastics, and phthalates, among others [3,4,5].

Phthalic acid esters (PAEs) or phthalates are synthetic compounds widely used in industry as color or aroma fixatives in food, perfumes, and plastic materials. They are mostly used as plasticizers in polymeric matrices of polyvinyl chloride, polyethylene terephthalate, polyvinyl acetate, and polyethylene to produce different items such as containers, bags, food packaging, hoses, pipes, seals, toys, medical devices, paintings, etc. [6,7,8]. Since phthalates are not chemically bound to polymeric plastics, when they come into contact with beverages, phthalates can migrate and contaminate them [7,9]. This has led to great concern regarding the presence of phthalates and their impact on human health. Phthalates are classified as endocrine disruptors and have been associated with various hormonal disorders, malformations, and cancer [10,11,12,13]. For this reason, health authorities have established the maximum permissible limits (MPLs) for the concentration of phthalates in beverages and foods, as well as data on tolerable daily intake (TDI) (Table 1). Di-ethylhexyl phthalate (DEHP) is the most widely compound used as a plasticizer, and it has been detected in alcoholic beverages, such as wine [11,12,13,14,15,16,17,18], beer [19,20,21], plum spirit [22], brandy [15,23], and tequila [24].

Although several reports demonstrate the presence of phthalates in commercial alcoholic beverages, to our knowledge, few reports indicate the origin of the contamination. For instance, Chatonnet et al. reported the presence of phthalates in wines and grape spirits, as well as the contamination sources and means of prevention. They concluded that various materials frequently used in wineries contained high concentrations of phthalates and that the epoxy resin coating used on vats was the major source of contamination [15]. Ye et al. found 6.22 μg L^−1^ DBP and 7.76 μg L^−1^ DEHP in bottles of Chinese beer and concluded that the high contents of these PAEs incorporated in polyvinyl chloride gaskets in the lids or liquid dispensers were the source of phthalate contamination in bottled beers during transportation and storage [20]. Balderas et al. documented the presence of phthalates in 295 tequila samples and reported that 78% of samples contained phthalates, among which DEHP was the most frequently found, ranging from 0.03 to 4.46 mg kg^−1^, but they did not report the source of contamination [24].

The objective of this study was to identify the origin and fate of PAEs in the tequila production process. For this, raw materials such as agave and water, as well as additives, lubricants, and neoprene seals were analyzed. Since DEHP was the only phthalate detected in quantifiable concentrations, mass balances for this compound were applied in each production stage of tequila (input and output).

## 2. Materials and Methods

### 2.1. Chemicals and Tequila

Di-ethylhexyl phthalate with purity up to 99.9% was used as standard (Sigma-Aldrich, St. Louis, MO, USA). HPLC-grade methanol (Tedia, Monterrey, NL, Mexico) was used for the preparation of standards, resuspension of dry extract, rinse of glassware, sampling and analysis. HPLC-grade dichloromethane (Sigma-Aldrich, St. Louis, MO, USA) was used as a DEHP extracting agent and glassware washing. Double-distilled water was used to rinse glassware. All materials and chemicals were analyzed to ensure that there were no DEHP contamination.

### 2.2. Tequila Factories and Sampling

Three tequila factories authorized by Tequila Regulatory Council (*Consejo Regulador del Tequila*, CRT), the certifying Mexican institution that guarantees the authenticity of the beverage, voluntarily agreed to participate in this study. These tequila factories were denominated according to their size as large, medium, and small. In each of these factories, three production batches were sampled. Each tequila production operation was analyzed by taking samples of all the materials that enter and leave the factory. Each sample consisting of 500 g or 500 mL, in the solid or liquid state, was stored in glass flasks, previously rinsed with double-distilled water, methanol, and dichloromethane to avoid contamination by PAEs. All glass materials were new, exclusively used for this experiment, and had never been put in contact with soap or detergent. The samples were kept at 4 °C until analysis.

Both raw agave and water were categorized as the raw materials, while cooked agave, agave juice, live must, dead must, and ordinary (first-distilled product) and rectified (second-distilled product) samples were collected as intermediate materials; white tequila was collected as the final product. Bagasse and vinasses were collected and classified as waste materials.

For the mass balance, the amount of DEHP in the input and output was determined in each production stage by multiplying the concentration of DEHP by the mass studied.

### 2.3. Additives, Lubricating Greases, and Neoprene Seals

Samples of additives and new and used lubricating greases from extractor mills were collected in all three tequila factories, as well as samples of neoprene used as a seal in the distillation tower, stills, and valves of the medium and small tequila factories. To quantify PAEs in all materials, 0.1 g of the sample was dissolved in dichloromethane, then dichloromethane was evaporated, and the dry extract was dissolved in 1 mL of methanol, filtered, and analyzed as described below.

### 2.4. Quantification of PAE

PAEs extraction followed by GC/MS analysis was performed using the GBT21911-2008 protocol [30], which is the standard technique accepted for the determination of phthalate esters in foods. This protocol has been validated and applied in a wide range of matrices for the analysis of PAEs, such as vegetable cans [31], plastic materials [31,32], alcoholic beverages [24], and microbiological culture media [33]. This protocol has been applied here for the first time in agave and vinasses and the recovery of DEHP was 88.5 to 108.3% and 82.9 to 107.4%, respectively (Supplementary Table S1). PAEs were recovered via a liquid–solid or liquid–liquid extraction according to the method reported by González-Castro et al. [31]. In brief, 10 g of solid material such as agave or 10 mL of liquid material such as water, juice, ordinary, rectified, must, tequila, or vinasses, and 10 mL of dichloromethane as the extracting agent were mixed. Dichloromethane was evaporated, producing a dry extract that was resuspended in 1 mL of methanol and analyzed in a gas chromatograph with a mass spectrometer detector GC/MS 7820A/5977E (Agilent Technologies, Inc., Palo Alto, CA, USA) by using an HP5-MS column (30 m × 250 µm × 0.25 µm; Agilent Technologies), with helium as a carrier gas at a flow rate of 1 mL min^−1^. For the analysis, 1 µL of the sample was injected by using an automatic injector 7683 (Agilent Technologies) in splitless mode. Chromatographic conditions used were according to Balderas-Hernández et al. [24]. The identity of compounds was determined by comparing the mass spectra obtained with the NIST 14 library (Gaithersburg, MD, USA), and the quantification was performed using commercial standards (Table 2). Quantification was performed in triplicate and the result analysis was based on average data.

### 2.5. Statistical Analysis

To identify significant differences between the amount of DEHP input and output in each production stage, a Student’s *t*-test was performed using the Excel Solver tool for Microsoft 365 MSO (ver. 2209) and *p* < 0.05 indicates statistical significance.

## 3. Results

**Figure 1:**
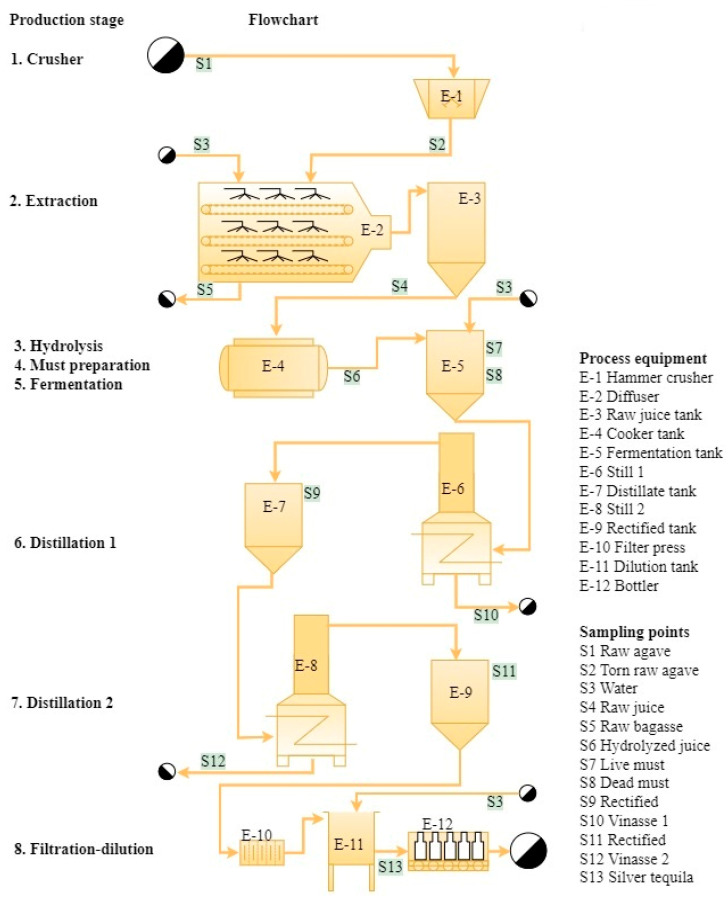
Flowchart of the large tequila factory, production stages, and sampling points.

**Figure 2:**
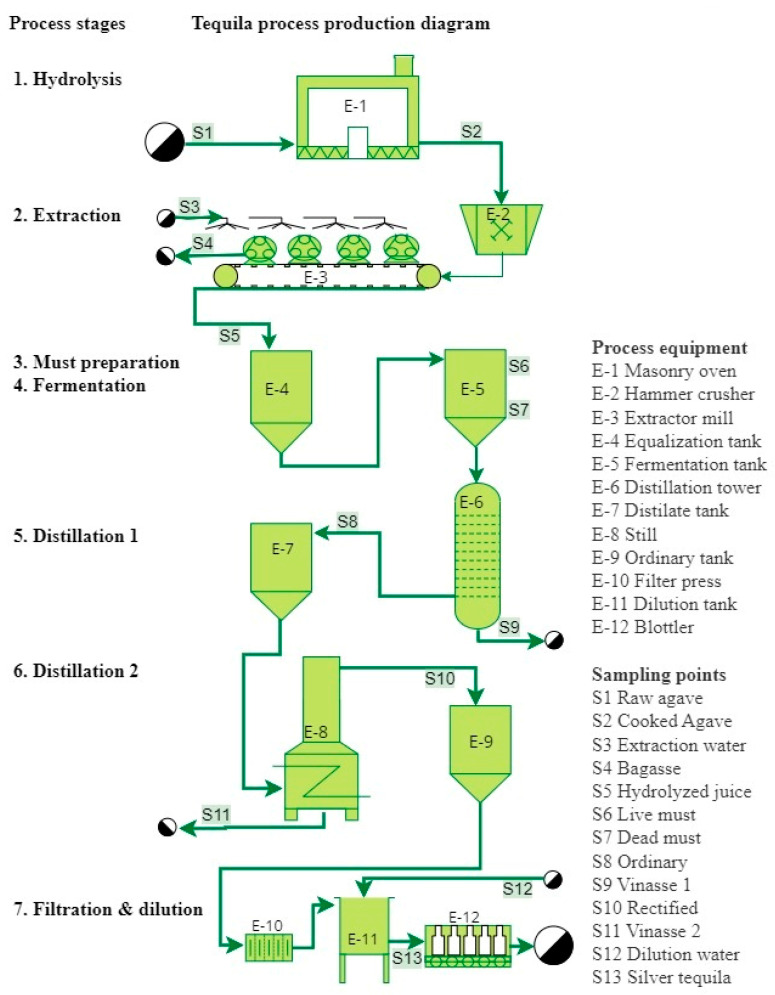
Flowchart of the medium tequila factory, production stages, and sampling points.

**Figure 3:**
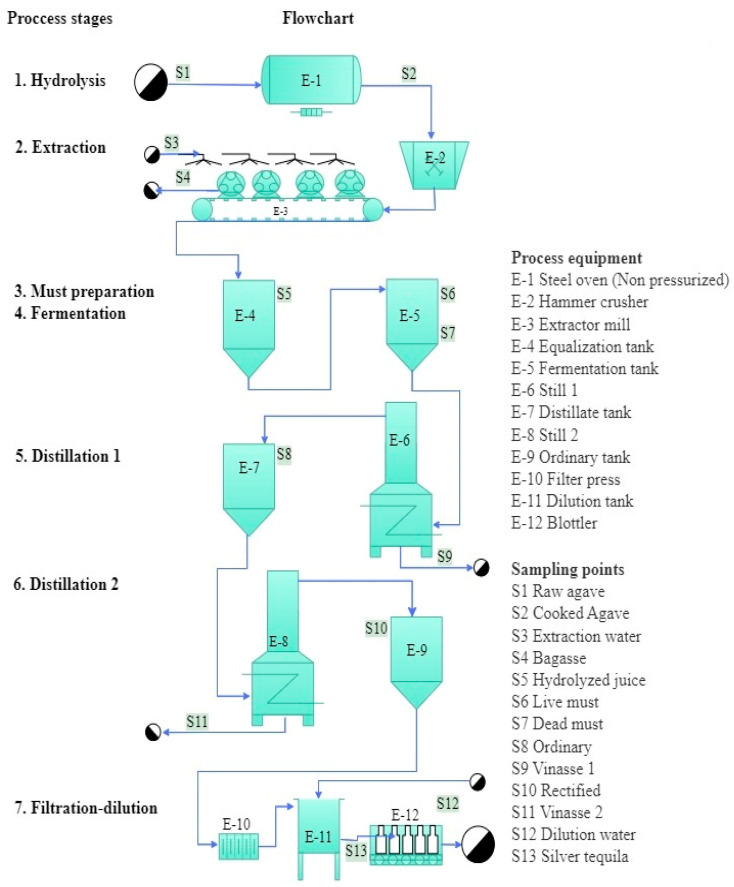
Flowchart of the small tequila factory, production stages, and sampling points.

The three tequila factories studied operated under a batch production system. Flowcharts of the tequila production process, production stages, and sampling points are shown in Figure 1, Figure 2 and Figure 3 and the main characteristics of factories and their production processes are summarized in Table 3. The process in the large factory was divided into eight production stages: crusher, extraction, hydrolysis, must preparation, fermentation, distillation 1, distillation 2, and filtration–dilution, while for the medium and small factories, the process was divided into seven production stages: hydrolysis, extraction, must preparation, fermentation, distillation 1, distillation 2, and filtration–dilution. The large factory extracts the inulin from the agave through a diffusion system (liquid–solid extraction), and the next step is thermal hydrolysis to release fermentable sugars. This system allows the processing of large quantities of raw agave up to 600 ton per batch (Figure 1). Whereas medium and small factories hydrolyze the inulin into the agave by cooking in a masonry oven (Figure 2) or stainless steel drying oven (Figure 3), with a processing capacity of 17 and 7.5 ton per batch, respectively, and the sugar extraction is carried out with roller mills. Another relevant difference observed was in the distilling step. The medium factory uses a 20 m distilling tower equipped with eight tubular segments united with 2 cm thick neoprene seals for the first distillation, followed by a still for the second distillation. Whereas the other factories use stills for both distillations.

Three batches from each tequila factory were sampled and analyzed to determine the presence of PAEs, but DEHP was the only one detected in quantifiable concentration, and therefore this study focused only on this compound. However, this methodology could be applied to the analysis of the DEP, DBP, BBP, and DINP in other alcoholic and non-alcoholic beverage production processes. DEHP profile of the production process of each factory is shown in Table 4. As observed, the presence of DEHP was detected in the two main raw materials: in agave at the concentrations of 0.012 ± 0.11, 0.079 ± 0.029, and 0.060 ± 0.021 mg kg^−1^, and in extraction water at 0.011 ± 0.001, 0.019 ± 0.014, and 0.014 ± 0.001 mg kg^−1^, in the large, medium, and small tequila factories, respectively. The highest concentrations of DEHP were detected in wastes such as bagasse at the concentrations of 0.215 ± 0.172, 0.231 ± 0.027, and 0.334 ± 0.023 mg kg^−1^, and vinasses at 0.012 ± 0.006, 0.122 ± 0.011, and 0.445 ± 0.016 mg kg^−1^. Whereas, rectified samples presented 0.059 ± 0.044, 0.190 ± 0.117, and 0.478 ± 0.382 mg kg^−1^ of DEHP in large, medium, and small factories, respectively. It can also be seen that the highest concentrations of DEHP in all materials occurred in the samples of medium and small factories, while the lowest concentrations were found in the large factory. The concentrations of DEHP in the final product of tequila were 0.045 ± 0.010, 0.238 ± 0.035, and 1.433 ± 0.480 mg kg^−1^, respectively.

**Figure 4:**
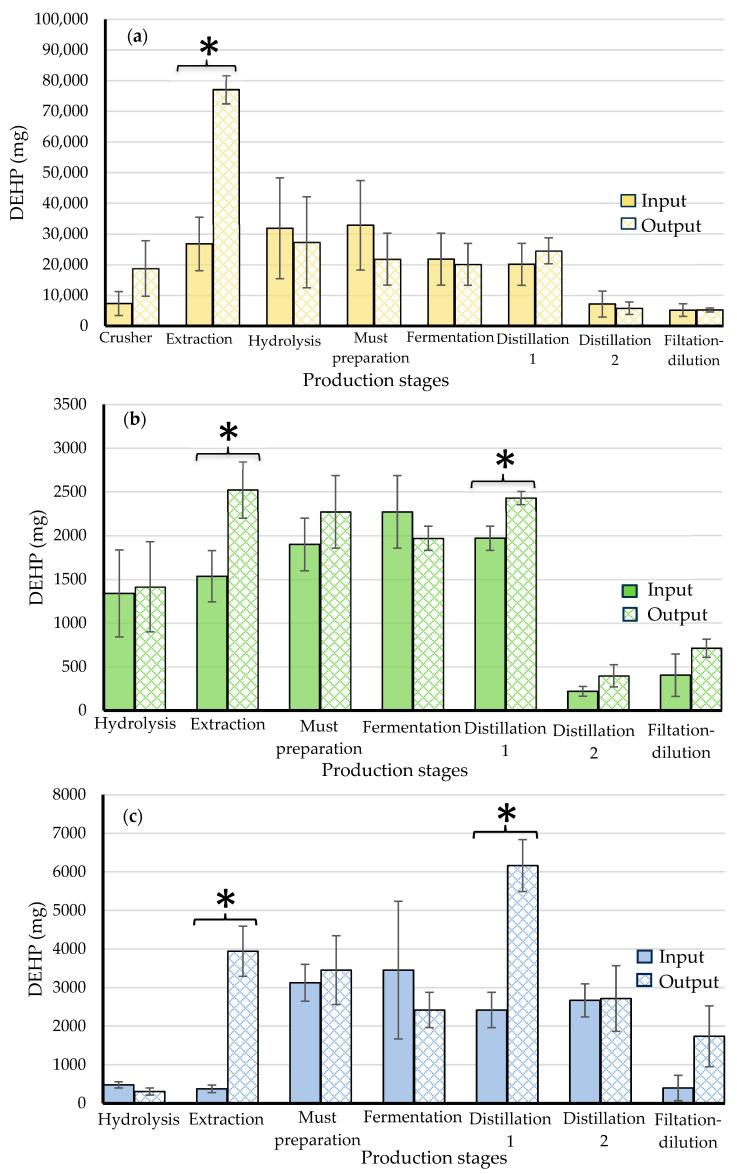
Comparison of the amount of input and output of DEHP at each stage of the production process for (**a**) large factory, (**b**) medium factory, and (**c**) small factory. Data represent the average and standard deviation of three independent batches. (*) Indicates a significant statistical difference between the input and the output for each production stage (*p* < 0.05).

Figure 4 shows the comparison of DEHP input and output at each stage of production for each factory. Figure 4a shows that in the large factory, only the extraction stage showed a significant difference in DEHP input and output, with significantly more DEHP coming out (77,050 ± 4629 mg) than going in (26,743 ± 8725 mg), which means that at this stage, there was a source of DEHP not attributable to the raw materials entering the operation; it could be attributed to the lubricating grease used in the roller mills. Figure 4a also shows that the amount of DEHP tends to be lower in the subsequent stages of the process, from 20,089 mg in the hydrolysis step to 2488 mg in the filtration and dilution step.

In the medium factory, extraction and distillation 1 stages showed that DEHP output was significantly higher than in the DEHP input, with 2522 ± 324 mg DEHP output and 1536 ± 293 mg input for the extraction stage; and 2431 ± 77 mg output and 1969 ± 138 mg input for the distillation 1 stage (Figure 4b). In the same way, Figure 4c indicates that in the small factory, the extraction and distillation 1 stages also showed that the amount of output DEHP was significantly greater than the input DEHP, with 3944 ± 648 mg output and 376 ± 97 mg input for the extraction stage; and 6165 ± 673 mg output and 2418 ± 458 mg input for the distillation 1 stage. This means that in these two factories, in the extraction stage, there was a source of DEHP attributable to the observed contact of the agave juice with the lubricating grease of the gears of the extraction mill, while in the distillation 1 stage, the increase can be attributed to the contact of the hot must with the neoprene seals of the distillation tower and stills.

**Figure 5:**
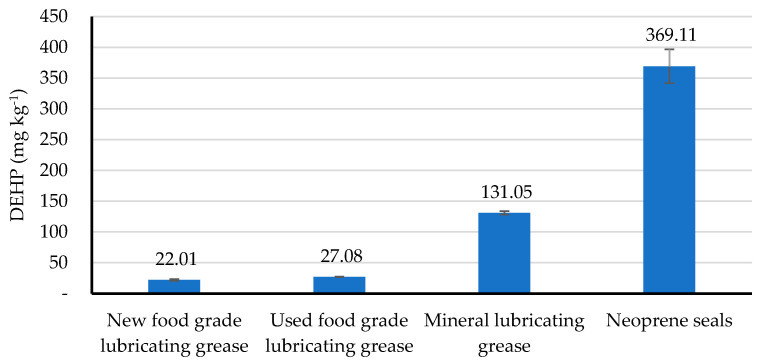
DEHP in lubricating greases and neoprene used as seals. Data represent the average and standard deviation (n = 2).

To support the theory that excess DEHP of the production stage outputs could come from lubricating greases or mechanical seals, both materials were analyzed and the results are shown in Figure 5. Mineral lubricating grease contained 131.05± 2.80 mg kg^−1^ DEHP, whereas the food grade grease had 22.01± 1.37 mg kg^−1^. DEHP in neoprene seals contained up to 369.11 ± 27.36 mg kg^−1^. Therefore, both materials were a source of DEHP contamination.

**Figure 6:**
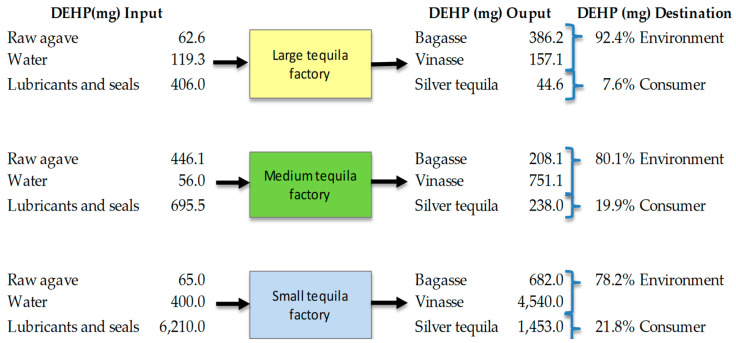
Flux of DEHP in the total mass balance during the production of 1000 L of tequila.

Di-ethylhexil phthalate left the process through two paths: in the residues (bagasse and vinasses), and in the final product. Figure 6 shows the total DEHP balance for the production of 1000 L of tequila in each factory. When this amount of tequila was produced by large, medium, and small factories, 543.3 mg, 959.2 mg, and 5222.6 mg of DEHP were released into the stillage treatment plants or the environment, while 44.6 ± 10 mg, 238.0 ± 35 mg, and 1452.7 ± 480 mg, respectively, could reach the customers through tequila consumption.

Seven additives authorized by the CRT to soften the aroma and color of tequila were analyzed (Table 5). DEHP concentration ranged from 0.324 to 2.863 mg L^−1^. Since they could be added to tequila in a ratio of 250 to 500 mL per 1000 L of tequila, they could contribute from 0.0002 to 0.0014 mg L^−1^ DEHP, which means that these additives do not represent a risk of DEHP contamination in the tequila.

## 4. Discussion

In this work, two sources of DEHP contamination were identified in the tequila production process. The first was the raw materials (agave and water). The presence of DEHP in agave is striking because it is a field-grown product. However, since 1997, it has been reported that phthalates are widely found in natural waters, solid sediments, and ecosystems [34], confirming the ubiquitous presence of phthalates. The DEHP concentration in the water used in the three factories ranged from 0.011 to 0.038 mg L^−1^; these values were higher than the maximum allowable concentration (MAC) for drinking water of 0.006 and 0.008 mg L^−1^ established by the US Environmental Protection Agency (US EPA) and the World Health Organization (WHO), respectively. The second source of DEHP was the lubricating greases (both food-grade and mineral-oil-based), and the neoprene seals used in the extraction equipment and distillation areas of medium and small factories, since they were in contact with juice and must during tequila production. Both materials contained a high concentration of DEHP, which can be transferred to tequila. DEHP transfer from neoprene is favored when this material is in close contact with non-polar or slightly polar solvents, at higher temperatures and acidic pH [35,36], such as must in distillation. Similarly to us, Jurica et al. determined the concentration of different phthalates in the five production stages of the plum spirit. They found that the stage with the greatest increase in the concentration of phthalates was the distillation. They found average values of 0.423 + 0.053 mg kg^−1^ DEHP, at a range of 0.016 to 1.638 mg kg^−1^, values close to those obtained in the medium and small tequila factories. Jurica et al. attributed the presence of phthalates to equipment and plastic containers, the acidity of the must, and the distillation [22].

In the study conducted by Chatonnet et al., phthalates were quantified in brandies and plastic materials used such as hoses, containers, and seals. They reported that the tank seal had the highest concentration of DEHP (29,684 mg kg^−1^) and it was the main source of contamination [15]. They also reported an average DEHP concentration and maximum values of 0.513 and 1.522 mg kg^−1^, respectively, in the brandies analyzed.

In the tequila factories analyzed here, two outputs of DEHP were identified. First, in the final product of tequila, DEHP of up to 0.045 ± 0.01 and 0.238 ± 0.035 mg kg^−1^ for tequila produced in the large- and medium-sized factories, respectively, was detected, which could reach consumers. These values are far lower than the MPLs by the health authorities. However, tequila produced in the small factory contained 1.433 ± 0.480 mg kg^−1^ DEHP, which represents a health risk for consumers, because the value is so close to the MPLs. The remaining DEHP left the process was through bagasse and vinasses residues. In some cases, the bagasse could be composted and used as fertilizer in the agave crop, while some vinasses go to treatment plants, where the removal of DEHP is not considered. As reported by Teil et al., water treatment plants cannot remove DEHP from residential and industrial wastewater effluent [37]. Lopez-Lopez et al. and Rodriguez-Romero et al. reported that most of the vinasses are thrown into bodies of water due to deficiencies in regulation [38,39].

In this work, the final product of the medium and small factories had 0.238 ± 0.035 and 1.433 ± 0.480 mg kg^−1^ of DEHP, respectively. Whereas Montevecchi et al. reported DEHP in a range of 0.190 to 2.640 mg kg^−1^ for brandies [23]. Balderas et al. reported an average DEHP concentration of 1.5 mg kg^−1^ in tequilas produced in 2013, a value similar to the average DEHP obtained in the small factory. While the DEHP averages obtained in 2017 and 2018, of 0.3 and 0.2 mg kg^−1^, respectively, were similar to those obtained in the medium factory [24].

The mass balance as a methodology to detect the DEHP contamination stages in an industrial process for the production of alcoholic beverages is proposed here for the first time. However, the PAEs quantitation requires a laborious sample processing and analysis through CG/MS, which can be an expensive technique for small companies and is not easy to use for online monitoring. 

## 5. Conclusions

The two main raw materials used for tequila production, agave and water, contain traces of DEHP, but the major source of contamination is due to the transference by the contact of the juice and musts with lubricating grease in the extraction stage and mechanic neoprene seals used at the joints of pipes, stills, and distillation tower. Whereas the additives do not represent a source of contamination. Only the DEHP contained in the tequila produced in the small factory could represent a health risk for the consumer, but if the consumption of this beverage is moderate, the risk is insignificant. In the three factories, the highest amount of DEHP comes out of the process through vinasses and bagasse. Identification of the contamination sources can help the tequila industry and others to take actions to reduce it, to protect the health of consumers and the environment, as well as to prevent circular contamination.

## Figures and Tables

**Table 1:** Maximum permissible limits for phthalates in non-fatty foods, including distilled spirits and tolerable daily intake [25,26,27,28,29].

Name	Abbreviation/CAS Number	Structure	Maximum Permissible Limit (mg kg^−1^)	Tolerable Daily Intake (mg kg^−1^ bw^−1^) *
Di-ethyl phthalate	DEP 84-66-2	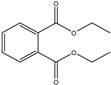	Not reported	Not reported
Di-butyl phthalate	DBP 84-74-2	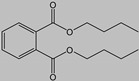	3.0	0.01
Benzyl butyl phthalate	BBP 85-68-7	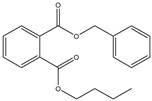	30.0	0.5
Di-ethylhexyl phthalate	DEHP 117-81-7	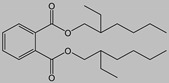	1.5	0.05
Di-isononyl phthalate	DINP 28553-12-0	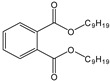	9.00	0.15

* bw: Body weight.

**Table 2:** Summary of information for PAEs identification.

Phthalate	Retention Time (min)	Ions for Identification	Ion for Quantification
DEP	8.72	149, 177, 76, 105	149
DBP	11.29	149, 104, 76, 223	149
BBP	15.41	149, 91, 206, 65	149
DEHP	17.79	149, 167, 57, 71	149
DINP	20.0–21.3	149, 71, 57, 293	149

**Table 3:** Main characteristics of tequila factories and their production processes.

Characteristics	Large Factory	Medium Factory	Small Factory
Location	Arandas, Jal. Mex.	Tequila, Jal. Mex.	Arenal, Jal. Mex.
Production (L yr^−1^)	More than 3 million	1–3 million	0.3–1 million
Crusher	Large equipment that uses food-grade lubricant grease	Small equipment that uses food-grade lubricant grease	Small equipment that uses mineral grease
Hydrolysis	Stainless steel pressurized container	Masonry oven	Stainless steel drying oven
Extraction	Diffuser (liquid–solid extraction with water and uncooked agave) uses food-grade lubricant grease	Roller mills that use food-grade lubricant grease	Roller mills that use mineral grease
Fermentation	Stainless steel tanks	Stainless steel tanks. Without the addition of yeast	Stainless steel tanks. Without the addition of yeast
Distillation 1	Still with teflon-covered neoprene seal (97 °C)	Distillation tower with neoprene seal (97 °C)	Still with neoprene seal (97 °C)
Distillation 2	Still with teflon-covered neoprene seal (97 °C)	Still with neoprene seal (97 °C)	Still with neoprene seal (97 °C)
Water supply	Total amount of water comes from a water well inside the grounds of the tequila factory.	Water for extraction and dilution comes from a city-managed water well. Dilution water is purchased from a water purification plant.	Water for extraction and dilution comes from a water well inside the grounds of the tequila factory. Dilution water is purchased from a water purification plant.
Waste treatment	Bagasse composting. Water treatment plant.	Vinasses are sent to a water treatment company.	Vinasses are sent to a water treatment company.

**Table 4:** DEHP concentration profile of the production process of each tequila factory.

Sample	Large Factory		Medium Factory		Small Factory	
	DEHP	S.D.	DEHP	S.D.	DEHP	S.D.
	(mg kg^−1^)		(mg kg^−1^)		(mg kg^−1^)	
Raw agave	0.012	0.011	0.079	0.029	0.06	0.021
Torn raw agave	0.031	0.026	ND	ND	ND	ND
Cooked agave	ND	ND	0.082	0.03	0.038	0.023
Bagasse	0.215	0.172	0.231	0.027	0.334	0.023
Extraction water	0.011	0.001	0.019	0.014	0.014	0.001
Raw juice	0.029	0.026	ND	ND	ND	ND
Hydrolysate	0.025	0.023	0.091	0.025	0.304	0.103
Live must	0.014	0.009	0.108	0.034	0.334	0.14
Dead must	0.013	0.007	0.094	0.011	0.233	0.068
Vinasse 1	0.012	0.006	0.122	0.011	0.445	0.16
Ordinary	0.04	0.041	0.079	0.036	1.117	0.434
Vinasse 2	0.011	0.007	0.042	0.02	1.214	1.126
Rectified	0.059	0.044	0.19	0.117	0.478	0.382
Dilution water	0.011	0.001	0.038	0.024	0.021	0.013
Silver tequila	0.045	0.01	0.238	0.035	1.433	0.48

ND: Not detected S.D.: Standard deviation (n = 3).

**Table 5:** DEHP concentration in the additives used in the softening of tequila.

Type of Additive	DEHP (mg L^−1^)	Contribution of DEHP per Liter of Tequila (mg L^−1^)
Wood aroma	0.575	0.0003
Cooked agave aroma	0.324	0.0002
Caramel coloring	1.849	0.0009
Oak extract	2.863	0.0014
Raw agave aroma	1.128	0.0006
Chocolate flavor	1.333	0.0007
Wood color	0.926	0.0005

## Data Availability

The data used to support the findings of this study can be made available by the corresponding author upon request.

## Supplementary Materials

The following supporting information can be downloaded at https://www.mdpi.com/article/10.3390/foods13020334/s1, Table S1: Percentage of DEHP recovered from agave and vinasses after the extraction with dichloromethane and analysis through GC/MS.
