# Supplementary material for: Tracing of Di-Ethylhexyl Phthalate in the Tequila Production Process
Source: Foods. 2024 Jan 20;13(2):334. doi: 10.3390/foods13020334 (PMC10814815; doi:10.3390/foods13020334)
Supplement: Supplementary file 1 [file foods-13-00334-s001.zip › foods-2660001-supplementary.pdf]

**Table S1.** Percentage of DEHP recovered from agave and vinasses after the extraction with dichloromethane and analysis by GC/MS.

| Quantity (µg) | Agave (%) | Quantity (µg) | Vinasses (%) |
|---|---|---|---|
| 1.75 | 108.3 | 0.07 | 82.9 |
| 3.50 | 103.9 | 0.175 | 107.4 |
| 7.00 | 88.5 | 0.70 | 94.9 |
